# The Prevalence of PTSD in a Forensic Psychiatric Setting: The Impact of Traumatic Lifetime Experiences

**DOI:** 10.3389/fpsyt.2022.843730

**Published:** 2022-04-27

**Authors:** Valeria Bianchini, Giovanna Paoletti, Roberta Ortenzi, Brunella Lagrotteria, Rita Roncone, Vincenza Cofini, Giuseppe Nicolò

**Affiliations:** ^1^Department of Life, Health and Environmental Sciences, University of L'Aquila, L'Aquila, Italy; ^2^Terzo Centro di Psicoterapia, Rome, Italy; ^3^DSMDP Asl Roma 5, REMS Castore, Contrada Colle Cisterna, Subiaco, Italy; ^4^Scuola di Terapia Cognitiva—APC, Rome, Italy; ^5^Scuola di Psicoterapia Cognitiva—SPC, Rome, Italy; ^6^Scuola Italiana Cognitivismo Clinico—SICC, Rome, Italy

**Keywords:** PTSD, trauma, forensic, offender, personality disorders

## Abstract

**Background:**

Several studies have identified traumatic history among forensic patients and its association with criminal behaviors and psychiatric diagnoses. Post-traumatic stress disorder (PTSD) is highly prevalent in forensic settings causing a serious deterioration of the primary psychiatric disorder.

**Aims:**

Our study aims to evaluate the prevalence of PTSD and the role of traumatic experiences and abuse in the development of severe psychiatric disorders in a sample of psychiatric offenders.

**Methods:**

Fifty-three patients admitted in Italian high intensity therapeutic facilities—the *Residenze per l'Esecuzione delle Misure di Sicurezza* (REMS)—were evaluated with the *Trauma Experience Checklist (TEC)* and the *Millon Clinical Multiaxal Inventory (MCMI-III)* to study lifetime traumatic memories and general psychopathology, respectively.

**Results:**

Preliminary findings show that about 41% (*N* = 22) of psychiatric offenders were affected by PTSD, often not due to a single episode but to multiple lifetime traumas. Therefore, lifetime traumatic experiences and specifically sexual abuse are significant risk factors for the development of a personality disorder, which is present in the 38% (*N* = 20) of the sample.

**Conclusions:**

The high PTSD prevalence and the strong association found between trauma and abuse with the development of a personality disorder emphasizes the importance of an early evaluation and intervention on traumatic experiences in this difficult population of psychiatric patients; in fact, the treatment of psychiatric offenders is actually vague and devoid of scientific evidence. Our results open up the perspective on the use of known and specific interventions for trauma, such as EMDR and Mindfulness.

## Introduction

People affected by Severe Mental Illness (SMI), such as schizophrenia spectrum disorders, bipolar disorders (BDs) and personality disorders (PDs), report elevated rates of traumatic experiences compared to the general population (1, 2).

Recent studies estimate a lifetime trauma exposure among SMI patients ranging between 16 and 98% (3). Grubaugh et al. (4) in a systematic review reported rates of trauma exposure ranging from 49 to 100%.

Trauma construct consists of one or more emotional, physical, or sexual events that can modify the subject's physical and psychological integrity (5). Several studies have found a strong association between early trauma and SMI; therefore, it is necessary to evaluate and treat traumatic memories in the clinical assessment of patients with serious psychiatric disorders (6).

More specifically, the presence of an early traumatic history among patients with SMI and PDs is associated with earlier onset of the mental disorder, more severe psychopathology (7, 8), higher medication doses (9), more serious and impaired global functioning (10), poorer treatment compliance (11), more substance abuse, higher relapse rate (12), and elevated risk of self-injurious and suicide (13, 14).

A history of traumatic experiences among individuals with SMI is further associated with a high risk of violent behavior, with a three times greater risk for SMI patients who have been exposed to any kind of trauma compared to patients without a traumatic history (15, 16): persistent early traumatic experiences have a negative and serious impact on brain development, mental health and aggressive behaviors in young people (17). Therefore, victimization is common among persons with severe mental disorders. Like other vulnerable populations (e.g., homeless persons, persons with developmental disabilities), persons with severe mental illness (SMI) are a particularly high-risk group (18). Symptoms associated with SMI, such as impaired reality testing, disorganized thought processes, impulsivity, and poor planning and problem solving, can compromise one's ability to perceive risks and protect oneself (19).

Other studies underlined the role of both interpersonal and collective traumas (e.g., natural disasters) to increase violent behaviors in youth (1, 20), with a cumulative effect of violence during the lifetime (5).

A few studies have assessed lifetime trauma exposure in patients affected by a PD; however, Battle et al. (21) in a longitudinal study reported that 73% of patients were exposed to a trauma in early age; in another review, as many as 90% of borderline PD patients were exposed to a traumatic experience (22). Other studies underlined that trauma exposure is higher in patients with borderline PD than in the general population (23, 24).

Different studies tested the role of Post Traumatic Stress Disorder (PTSD) as a mediator between trauma exposure and violence in youth but revealed inconsistent and rather poor evidence (25). Every-Palmer et al. (26) analyzing a sample of young offenders, showed a strong relationship between PTSD and violence perpetration with gender differences (being stronger in male than in female).

A lot of studies in UK medium secure units have found that between a third to over half of inpatients experienced PTSD in relation to their own violent offense (27). Physical abuse constituted the most common trauma, experienced by 75% of participants (28, 29).

This association between traumatic lifetime episodes, violent behavior and a diagnosis of serious mental illness, greatly complicate the treatment and management of adults with SMI (6, 30); it is known that childhood trauma is a recognized obstacle to treatment response in individuals with SMI (16, 31).

The treatment guidance on psychosis and schizophrenia of the National Institute of Clinical Excellence (32) recommends that all patients are routinely screened for trauma symptoms. The clinical aftermath of inaccurate PTSD identification and contraindicated treatment has been found to inadvertently lead to patient harm, further highlighting the need to identify and assess trauma in forensic populations (33, 34).

The 81/2014 Italian Law marked a historical change: the definitive closure of forensic psychiatric hospitals that have been replaced by a new pathway of care that involves high security but also intensively therapeutic units, the so-called REMS (Residenze per la Esecuzione della Misura di Sicurezza). These innovative settings admit psychiatric offenders with SMI that are treated according to a new recovery–rehabilitation approach (33, 35).

In this study we aim to analyze the prevalence of PTSD symptoms and the impact of lifetime trauma exposure in the development of severe psychiatric disorders (specifically PDs) in a sample of offenders admitted to REMS.

## Methods

The study was approved by the Local Ethic Committee (Comitato Etico Lazio 1).

### Subjects

Our sample consisted of 53 male patients consecutively admitted to one of the four Italian forensic and rehabilitation mental health services of the Rome 5 Department of Mental Health—the *Residenze per l'Esecuzione delle Misure di Sicurezza* (REMS)—recruited from July 2017 to December 2019. Written informed consent was obtained from each participant who had voluntarily agreed to participate. We protected the privacy and anonymity of the individuals involved.

In 2015 all six forensic psychiatric hospitals in Italy were replaced by small-scale and high intensively therapeutic units—the REMS: these innovative mental health settings, in accordance with the recent process of deinstitutionalisation, offer a new recovery–rehabilitation approach with the ultimate objective of reintegrating people with mental disorders and a criminal history back into the community (35). People admitted to an REMS, received a multidisciplinary and complete intervention in order to treat with pharmacology and not approaches psychiatric disorders, to improve the compliance, insight and social reintegration.

The eligibility criteria were: (a) lifetime violent episodes and criminal behavior; (b) age between 18 and 65 years; (c) a lifetime history of a SMI and/or PDs according to DSM-5 criteria; (d) ability to provide informed consent; and (e) the absence of a cognitive deterioration.

Patients were excluded if there was evidence of cognitive deficit (IQ < 70) and/or of comorbid neurological diseases. No participant reported a previous diagnosis of PTSD and any specific treatment for traumatic experiences.

A team of psychiatrists and clinical psychologists assessed patients to obtain details about trauma history and presence of PTSD using the *Trauma Experience Checklist (TEC)* (36) and specific items of the *Millon Clinical Multiaxal Inventory (MCMI-III)* (37), respectively.

- the **Traumatic experience checklist (TEC)** (36) is a self-reported checklist addressing 29 types of potentially traumatic events lifetime. It is a reliable and valid self-reported checklist that can be used in both clinical practice and research. It consists of a cumulative score and four dimensions: emotional neglect, emotional abuse, physical abuse, and sexual abuse. The TEC has demonstrated good convergent validity, being associated with alleged reports and official records of traumatic experiences (38).
- the **Millon clinical multiaxial inventory-III (MCMI-III)** (37) is a true-false self-report inventory consisting of 175 items grouped into 24 clinical scales arranged into four distinct categories: clinical personality patterns, severe personality pathology, clinical syndromes, and severe clinical syndromes. Retest intervals between 5 days and 4 months have provided a median value of reliability across the personality disorder (PD) scales of *r* = 0.78, ranging from 0.58 to 0.93 (39).

### Statistical Analyses

Descriptive analyses were reported as mean or standard deviation (SD) or median and Interquartile range (IQR) for continuous variables and, as frequency and percentage for categorical variables. Two-sample Wilcoxon rank-sum (Mann-Whitney) test or Chi square test were used to investigate differences between patients with or without a personality disorder (PD).

A logistic regression model for univariate and multivariable analysis was run to investigate the association between personality disorder (PD) (dependent variable) and the following covariates: TEC (score); emotional abuse (score); physical abuse (score); sexual abuse (score); PTSD (yes/no), as independent variables. Unadjusted odds ratio (OR) and adjusted odds ratio (ORadj) with 95%CI were reported.

The statistical package STATA14 MP, was used for all analyses; a *p* < 0.05 (2-tailed) was used for statistical significance.

## Results

Our sample consists of 53 male psychiatric patients with different criminal offenses; their mean age was 37.4 years (SD = 5.56 years) and their educational level was 9.7 years (SD = 3.4 years).

The majority of patients (43%; *N* = 23) had a schizophrenic spectrum disorder, 13% were affected by bipolar disorder (*N* = 7), and 38% (*N* = 20) had a diagnosis of personality disorders, with antisocial (*N* = 7) and borderline personality disorder (*N* = 6) being the most common ones. The mean age of psychiatric disorder onset was 22.04 years (SD = 5.9 years), with 16.71 years (SD = 3.2 years) of illness. 72% (*N* = 38) of the sample had a comorbid substance abuse disorder. The average length of admission was 13.4 months (SD =2.6). No patient had been scheduled or segregated during admission. 51% of the sample had personal injury in the family setting as reason for reclusion. The descriptive and clinical features of participants are reported in Table 1.

**Table 1:** Socio-demographic, forensic and clinical data of sample (*N* = 53).

	***N* (%)**	**Mean (SD)**
Mean age (years)		37.4 ys (SD = 5.56)
Educational level (years)		9.7 ys (SD = 3.42)
Primary diagnosis		
Schizophrenia	23 (43%)	
Personality disorders	20 (38%)	
Bipolar disorder	7 (13%)	
Others	3 (6%)	
Mean age of psychiatric disorder (years)		22.04 ys (SD = 5.93)
Mean age of psychiatric Illness (years)		16.71 ys (SD = 3.24)
Average length of admission (months)		13.4 months (SD = 2.61)
History of substance abuse		
Yes	38 (72%)	
No	15 (28%)	

Mean value of TEC total score was 14.9 (SD = 7.9) with a mean of 6.7 (DS = 4.3) for Emotional Abuse, 4.2 (SD = 3.6) for Physical abuse (score) and 2.7 (SD = 3.2) for the Sexual abuse (score). 41% (*N* = 22) (95%CI: 29–55%) of our forensic sample reported PTDS with a pathological score to Millon Clinical Multiaxial Inventory-II > 75.

Among patients with PD (20/53: 95%CI: 25–52%), the univariate analysis showed a TEC mean score significantly higher than subjects without PD (*p* = 0.0002), and Sexual abuse score was significantly higher (*p* = 0.0075), evidencing that Sexual abuse was a factor risk for PD (OR = 1.31; 95% CI: 1.08–1.59). Among patients with PD, 12 subjects (60%) were affected by PDTS, then there was a significant association between PD and PDTS (*p* ≤ 0.033), and PDTS was a risk factor for PD (OR = 3.45; 95% CI: 1.08–11.03), as reported in Table 2.

**Table 2:** Factors related to personality disorder (univariate analysis).

	**Disturbo della personalità**		*P* ^*^	OR^**^	**95%CI**
	**Yes**	**No**			
	**Median (IQR) or *n* (%)**	**Median (IQR) or *n* (%)**			
Tect totale (score)	17.5 (20)	16 (21)	0.1737	1.04	0.97–1.13
Abuso emotivo (score)	8.5 (4.5)	6 (10)	0.2907	1.09	0.96–1.25
Fisico (score)	7 (7)	2 (8)	0.0613	1.17	0.99–1.39
Sessuale (score)	3 (9)	0 (3)	**0.0075**	1.31	1.08–1.59
Trauma (yes)	12 (60%)	8 (40%)	**0.033**	3.45	1.08–11.03

* *Two-sample Wilcoxon rank-sum (Mann-Whitney) test or Chi square test*.

** *Univariate logistic regression model*.

The multivariable analysis that controlled for all investigated factors, has found that the Sexual abuse was a significant risk factor for a personality disorder (OR = 1.27; 95% CI: 1.00–1.61) as reported in Table 3.

**Table 3:** Factors related to personality disorder (multivariable analysis).

**Indipendent variables**	**Coefficients**	**OR**	***p***	**95%CI**
Tec totale (score)	−0.037	0.96	0.474	0.87–1.07
Emotional Abuse (score)	0.0495	1.05	0.549	0.89–1.23
Physical abuse (score)	0.0250	1.02	0.815	0.89–1.23
Sexual abuse (score)	0.241	1.27	**0.046**	**1.00–1.61**
Trauma (yes)	1.046	2.84	0.164	0.65–12.41

*Cox-Snell/ML's R^2^: 0.914; R^2^ Cox-Snell/ML: 0.194; Cragg-Uhler/Nagelkerke's R^2^: 0.264*.

## Discussion

This is the first Italian study to examine the prevalence of PTSD among a forensic sample and the association between PTSD symptoms, different types of abuse (emotional, physical and sexual) and primary DSM-5 diagnosis.

We performed two sets of analyses: with the first set, we examined the prevalence rates of PTSD using *Millon Clinical Multiaxial Inventory-III* (MCMI-III) among our 53 forensic inpatients admitted consecutively in the *Castore* REMS, Subiaco (RM), Lazio, Italy.

PTSD among forensic patients is common but often unaddressed (17, 40). Several evidence underlines traumatic experiences frequently present with comorbid SMI and PDs, and in turn can be associated with offending. Traumatic events play a central role as they seem to impair the ability of mentalizing or symbolizing emotions (41).

Among a UK male prison sample, prevalence of PTSD was found to range from 0.1 to 27%, showing that the identification of traumatic experiences in this population is highly variable (17). Another UK study among a male medium secure service sample found that no patient had a diagnosis of PTSD, but 93% had a traumatic lifetime experience (42). Our analyses showed a prevalence of PTSD in the forensic setting of 41% (*N* = 22), suggesting a pervasive and persistent under-identification of the impact of trauma and trauma-related psychiatric illness in forensic settings.

A few studies have addressed the developmental mechanisms of trauma and violence in psychiatric offenders, but it is known that post-traumatic symptoms in adult veterans predict later violent behavior (43).

The few studies that have examined traumatic experiences in forensic populations report trauma rates that are significantly higher than those reported in the general population, and also similar to non-forensic psychiatric patients affected by SMI and/or PDs. Garieballa et al. (44) analyzed 31 forensic inpatients and found that all participants reported at least one lifetime trauma.

Our results show that 67% (*N* = 54) of forensic patients had been exposed to a traumatic event in their lifetime, either in childhood or in adulthood: 62% (*N* = 33) reported emotional abuse, 27% childhood physical abuse, and 5% (*N* = 3) experienced a sexual abuse. These data are in line with those reported by Spitzer et al. (30) in a German maximum-security sample, who found that 64% of patients with SMI and PD had experienced one lifetime trauma. Moreover, a UK study comparing forensic patients vs. general psychiatric inpatients found higher rates of trauma exposure among the forensic group (42).

Our conclusions are similar to a recent Dutch study that analyzed 436 forensic psychiatric patients and found that 67% had experienced trauma in childhood and 36.5% in adulthood (45). Jones (46) reported that 55% of male population at Rampton HSH had experienced physical abuse, 36% sexual abuse, 61% emotional abuse, 51% emotional neglect and 26% physical neglect.

The second step of our study is the identification of traumatic experiences like risk factor for the development of a personality disorder in a sample of psychiatric offenders.

Fifty-five percent of our forensic sample reporting PTSD was affected by a PD with a significant association (*p* ≤ 0.033). The univariate analyses of PDs and traumatic experiences showed that trauma (TEC total score) and sexual abuse (OR 1.3 – IC 1.1–1.6) are risk factors for the development of PDs.

Only a few studies investigated lifetime trauma exposure in people with PDs: Batle et al. (21) reported a childhood trauma in 73% of patients with a PD diagnosis, and other studies on adult trauma and PDs have found higher rates than the general population, particularly in the case of borderline personality disorder (23). In our sample, the most common PDs were antisocial (*N* = 7) and borderline personality disorder (*N* = 6).

Our study has several methodological limitations, that hereby acknowledged. In particular, one limitation is the relatively small sample size, which is due to the low number of possible admissions in REMS (maximum 20 inpatients per facility) and the low turnover of patients who remain interned even for years. A second limitation is the use of self-reporting assessment instruments, which has been done in order to guarantee patients' confidentiality in a rather difficult sample.

Another limitation is the fact that we did not consider trauma that occurred during forensic patients' admission to a secure unit; several studies have found that hospitalization in custodial psychiatric and non-psychiatric settings can involve exposure to several traumatic experiences including physical and sexual abuse, seclusion and/or segregation, involuntary admission, forced medication and physical restraint (47, 48).

In conclusion, our results direct to important considerations in forensic setting: first, the significant relationship between traumatic lifetime experiences and the development of Personality Disorders, one of the most common and difficult diagnosis in forensic settings. The second point is the high prevalence of PTSD among psychiatric offenders: one of the most important clinical problem in forensic settings is the lack of evidence on therapeutic and rehabilitative treatments; so we hope that our findings will underline the importance of identification and intervention on post-traumatic maybe indirectly to reduce the risk of violence and new crimes among psychiatric offenders.

## Data Availability Statement

The original contributions presented in the study are included in the article/supplementary material, further inquiries can be directed to the corresponding author/s.

## Ethics Statement

The studies involving human participants were reviewed and approved by Comitato Lazio 1. The patients/participants provided their written informed consent to participate in this study.

## Author Contributions

VB, GP, and GN designed and wrote the study. VC elaborated statistical data. RO and BL recruited patients and collected data. All authors contributed to the article and approved the submitted version.

## Conflict of Interest

The authors declare that the research was conducted in the absence of any commercial or financial relationships that could be construed as a potential conflict of interest.

## Publisher's Note

All claims expressed in this article are solely those of the authors and do not necessarily represent those of their affiliated organizations, or those of the publisher, the editors and the reviewers. Any product that may be evaluated in this article, or claim that may be made by its manufacturer, is not guaranteed or endorsed by the publisher.

## References

[1] Spidel A Lecomte T Greaves C Sahlstrom K Yuille JC. Early psychosis and aggression: predictors and prevalence of violent behaviour amongst individuals with early onset psychosis. Int J Law Psychiatry. (2010) 33:171–6. 10.1016/j.ijlp.2010.03.007 20546896

[2] Mueser KT Lu W Rosenberg SD Wolfe R. The trauma of psychosis: posttraumatic stress disorder and recent onset psychosis. Schizophr Res. (2010) 116:217–27. 10.1016/j.schres.2009.10.025 19939633

[3] Sin J Spain D Furuta M Murrells T Norman I. Psychological interventions for post-traumatic stress disorder (PTSD) in people with severe mental illness. Cochrane Database Syst Rev. (2017) 1:CD011464. 10.1002/14651858.CD011464.pub2 28116752 PMC6464771

[4] Grubaugh AL Zinzow HM Paul L Egede LE Frueh BC. Trauma exposure and posttraumatic stress disorder in adults with severe mental illness: a critical review. Clin Psychol Rev. (2011) 31:883–99. 10.1016/j.cpr.2011.04.003 21596012 PMC3148295

[5] American Psychiatric Association. Diagnostic and Statistical Manual of Mental Disorders, Fifth Edition (DSM-5). Arlington: American Psychiatric Association (2013).

[6] Bruce M Laporte D. Childhood trauma, antisocial personality typologies and recent violent acts among inpatient males with severe mental illness: Exploring an explanatory pathway. Schizophr Res. (2015) 162:285–90. 10.1016/j.schres.2014.12.028 25636995

[7] Maniglio R. Severe mental illness and criminal victimization: a systematic review. Acta Psychiatr Scand. (2009) 119:180–91. 10.1111/j.1600-0447.2008.01300.x 19016668

[8] Pompili M Giordano G Luciano M Lamis DA Del Vecchio V Serafini G. Unmet needs in schizophrenia. CNS Neurol Disord Drug Targets. (2017) 16:870–84. 10.2174/1871527316666170803143927 28782490

[9] Schneeberger AR Muenzenmaier K Castille D Battaglia J Link B. Use of psychotropic medication groups in people with severe mental illness and stressful childhood experiences. J Trauma Dissoc. (2014) 15:494–511. 10.1080/15299732.2014.903550 24678974

[10] Pagura J Stein MB Bolton JM Cox BJ Grant B Sareen J. Comorbidity of borderline personality disorder and posttraumatic stress disorder in the U.S. population. J Psychiatric Res. (2010) 44:1190–8. 10.1016/j.jpsychires.2010.04.016 20537660 PMC4209725

[11] Lecomte T Spidel A Leclerc C MacEwan GW Greaves C Bentall RP. Predictors and profiles of treatment non-adherence and engagement in services problems in early psychosis. Schizophr Res. (2008) 102:295–302. 10.1016/j.schres.2008.01.024 18295458

[12] Gairns S Alvarez-Jimenez M Hulbert C McGorry P Bendall S. Perceptions of clinicians treating young people with first-episode psychosis for post-traumatic stress disorder. Early Interven Psychiatry. (2015) 9:12–20. 10.1111/eip.12065 23802596

[13] O'Hare T Shen C Sherrer M. Lifetime trauma and suicide attempts in older clients with severe mental illness. Soc Work Ment Health. (2018) 16:505–17. 10.1080/15332985.2018.1444694 24282033

[14] Subica AM Claypoole KH Wylie AM. PTSD'S mediation of the relationships between trauma, depression, substance abuse, mental health, and physical health in individuals with severe mental illness: evaluating a comprehensive model. Schizophr Res. (2012) 136:104–9. 10.1016/j.schres.2011.10.018 22104139

[15] Nikulina V Widom CS Czaja S. The role of childhood neglect and childhood poverty in predicting mental health, academic achievement and crime in adulthood. Am J Community Psychol. (2011). 48:309–21. 10.1007/s10464-010-9385-y 21116706 PMC7197378

[16] Seow LSE Ong C Mahesh MV Sagaya V Shafie S Chong SA. A systematic review on comorbid post-traumatic stress disorder in schizophrenia. Schizophr Res. (2016) 176:441–51. 10.1016/j.schres.2016.05.004 27230289

[17] Baranyi G Cassidy M Fazel S Priebe S Mundt AP. Prevalence of posttraumatic stress disorder in prisoners. Epidemiol Rev. (2018) 40:134–45. 10.1093/epirev/mxx015 29596582 PMC5982805

[18] Wendy MM Albers DP Roeg K Yolanda N Jaap van Weeghel IM Bongers B. Profiling of victimization, perpetration, and participation: a latent class analysis among people with severe mental illness. PLoS One. (2018) 13:e0208457. 10.1371/journal.pone.0208457 30500851 PMC6268008

[19] Desmarais SL Van Dorn RA Johnson KL Grimm KJ Douglas KS Swartz MS. Community violence perpetration and victimization among adults with mental illnesses. Am J Public Health. (2014) 104:2342–9. 10.2105/AJPH.2013.301680 24524530 PMC4133297

[20] Papanastassiou M Waldron G Boyle J Chesterman LP. Post-traumatic stress disorder in mentally ill perpetrators of homicide. J Forensic Psychiatry Psychol. (2004) 15:66–75. 10.1080/1478994031000163041925546989

[21] Battle CL Shea MT Johnson DM Yen S Zlotnick C Zanarini MC. Childhood maltreatment associated with adult personality disorders: findings from the collaborative longitudinal personality disorders study. J Pers Disord. (2004) 18:193–211. 10.1521/pedi.18.2.193.32777 15176757

[22] MacIntosh HB Godbout N Dubash N. Borderline personality disorder: Disorder of trauma or personality, a review of the empirical literature. Can Psychol. (2015) 56:227–41. 10.1037/cap0000028

[23] de Aquino LF Queiroz FH Neri AML Aguiar MC. Borderline personality disorder and sexual abuse: A systematic review. Psychiatry Res. (2018) 262:70–7. 10.1016/j.psychres.2018.01.043 29407572

[24] Slotema CW Wilhelmus B Arends LR Franken IH. Psychotherapy for posttraumatic stress disorder in patients with borderline personality disorder: a systematic review and meta-analysis of its efficacy and safety. Eur J Psychotraumatol. (2020) 11:1796188. 10.1080/20008198.2020.1796188 33062206 PMC7534189

[25] Clark CJ Spencer RA Everson-Rose SA Brady SS Mason SM Connett JE. Dating violence, childhood maltreatment, and BMI from adolescence to young adulthood. Pediatrics. (2014) 134:678–85. 10.1542/peds.2014-1179 25201793 PMC4179102

[26] Every-Palmer S Flewett T Dean S Hansby O Colman A Weatherall M Bell E. Eye movement desensitization and reprocessing (EMDR) therapy for posttraumatic stress disorder in adults with serious mental illness within forensic and rehabilitation services: a study protocol for a randomized controlled trial. Trials. (2019) 20:642. 10.1186/s13063-019-3760-2 31753032 PMC6868700

[27] Scott M. TIC in The State Hospital. In: Presentation at the High Secure Hospitals Psychology Conference, Merseyside (2017).

[28] Olff M Amstadter A Armour C Birkeland MS Bui E Cloitre M. A decennial review of psychotraumatology: what did we learn and where are we going? Eur J Psychotraumatol. (2019) 10:1672948. 10.1080/20008198.2019.1672948 31897268 PMC6924542

[29] McKenna G Jackson N Browne C. Trauma history in a high secure male forensic inpatient population. Int J Law Psychiatry. (2019) 66:101475. 10.1016/j.ijlp.2019.101475 31706394

[30] Spitzer C Dudeck M Liss H Orlob S Gillner M Freyberger HJ. Posttraumatic stress disorder in forensic inpatients. J Forensic Psychiatry. (2001) 12:63–77. 10.1080/09585180121757 12689627

[31] Paterniti S Sterner I Caldwell C Bisserbe JC. Childhood neglect predicts the course of major depression in a tertiary care sample: a follow-up study. BMC Psychiatry. (2017) 17:113. 10.1186/s12888-017-1270-x 28351403 PMC5370474

[32] National Institute for Health Care Excellence (NICE). Psychosis and Schizophrenia in Adults: Prevention and Management. (2014). Available online at: https://www.nice.org.uk/guidance/cg178/resources/psychosis-and-schizophrenia-in-adults-prevention-and-management-pdf-35109758952133 32207892

[33] Bianchini V Cofini V Curto M Lagrotteria B Manzi A Navari S. Dialectical behaviour therapy (DBT) for forensic psychiatric patients: an Italian pilot study. Crim Behav Mental Health. (2019) 29:2102. 10.1002/cbm.2102 30648303

[34] Lanius RA Frewen PA Tursich M Jetly R McKinnon MC. Restoring large-scale brain networks in PTSD and related disorders: a proposal for neuroscientifically-informed treatment interventions. Eur J Psychotraumatol. (2015) 6:27313. 10.3402/ejpt.v6.27313 25854674 PMC4390556

[35] Casacchia M Malavolta M Bianchini V Giusti L Di Michele V Giosue P. Closing forensic psychiatric hospitals in Italy: a new deal for mental health care?. Riv Psichiatr. (2015) 50:199–209. 10.1708/2040.22158 Italian. 26489069

[36] Nijenhuis ERS Van der Hart O Kruger K. The psychometric characteristics of the traumatic experiences questionnaire (TEC): first findings among psychiatric outpatients. Clin Psychol Psychotherapy. (2002) 9:200210. 10.1002/cpp.332

[37] Millon T. Disorders of Personality: Introducing a DSM/ICD Spectrum From Normal to Abnormal. 3rd edition. Hoboken: Wiley (2011).

[38] Craparo G Schimmenti A Caretti V. Traumatic experiences in childhood and psychopathy: a study on a sample of violent offenders from Italy. Eur J Psychotraumatol. (2013) 4:21471. 10.3402/ejpt.v4i0.21471 24371511 PMC3871837

[39] Strack S Millon T. Contributions to the dimensional assessment of personality disorders using millon's model and the millon clinical multiaxial inventory (MCMI-III). J Pers Assess. (2007) 89:56–69. 10.1080/00223890701357217 17604534

[40] Prins SJ. Prevalence of mental illnesses in US state prisons: a systematic review. Psychiatr Serv. (2014) 65:862–72. 10.1176/appi.ps.201300166 24686574 PMC4182175

[41] Gabbard GO. Psychodynamic Psychiatry in Clinical Practice. 4th ed. (2014). Washington, DC: American Psychiatric Pub Inc.

[42] Sarkar J Mezey G Cohen A Singh SP Olumoroti O. Comorbidity of posttraumatic stress disorder and paranoid schizophrenia: a comparison of offender and non-offender patients. J For Psychiatry Psychol. (2005) 16:660–70. 10.1080/14789940500279836

[43] Elbogen EB Johnson SC Wagner HR Sullivan C Taft CT Beckham JC. Violent behaviour and post-traumatic stress disorder in US Iraq and Afghanistan veterans. Br J Psychiatry. (2014) 204:368–75. 10.1192/bjp.bp.113.134627 24578444 PMC4006087

[44] Garieballa SS Schauer M Neuner F Saleptsi E Kluttig T Elbert T. Traumatic events, PTSD, and psychiatric comorbidity in forensic patients—assessed by questionnaires and diagnostic interview. Clin Pract Epidemiol Mental Health. (2006) 2:7. 10.1186/1745-0179-2-7 16595005 PMC1488838

[45] Bohle A de Vogel V. Gender differences in victimization and the relation to personality disorders in forensic psychiatry. J Aggress Maltreatment Trauma. (2017) 26:411–29. 10.1080/10926771.2017.1284170

[46] Jones L. Overview of trauma informed care at rampton. In: Presentation at the High Secure Hospitals Psychology Conference, Merseyside (2017).

[47] Paksarian D Mojtabai R Kotov R Cullen B Nugent KL Bromet EJ. Perceived trauma during hospitalization and treatment participation among individuals with psychotic disorders. Psychiatric Serv. (2014) 65:266–9. 10.1176/appi.ps.201200556 24492906 PMC4039016

[48] Wykes T Haro JM Belli SR Obradors-Tarragó C Arango C Ayuso-Mateos JL. Mental health research priorities for Europe. Lancet Psychiatric. (2015) 2:1036–42. 10.1016/S2215-0366(15)00332-6 26404415

